# Neurobiosensory and Visual Patterns of Attentional and Emotional Functioning in Children Using Machine Learning: Cross-Sectional Study

**DOI:** 10.2196/90041

**Published:** 2026-08-10

**Authors:** Ana Paula Azevedo, Andreia Sousa, Marta Evangelista, Ana Veloso, M Graça Pereira

**Affiliations:** 1 Neurosensorial Center Braga Portugal; 2 Institute of Systems and Computer Engineering Innovation (INOV)-Systems and Computing Engineering Institute (INESC), Lisboa Higher Institute of Engineering of Porto Porto Portugal; 3 Research Centre in Psychology School of Psychology University of Minho Braga Portugal

**Keywords:** attentional functioning, eye-tracking, machine learning, real-time multimodal capture, risk profiles, screening-support, support neurovisual assessment

## Abstract

**Background:**

Attentional functioning in childhood emerges from the interaction between cognitive, behavioral, and emotional processes. Children with these vulnerabilities frequently exhibit executive function deficits, including working memory, inhibitory control, cognitive flexibility, and processing speed, which compromise the ability to regulate attention, plan, and organize tasks, and modulate emotional responses, highlighting the need for screening approaches that integrate multiple dimensions of child functioning.

**Objective:**

This study aimed to develop and evaluate an automated, real-time, and simultaneous multimodal screening approach capable of identifying individualized cognitive-emotional-attentional profiles and behavior patterns in children through the integration of oculomotor, emotional, and neuropsychological measures, supporting earlier detection and personalized interventions.

**Methods:**

A cross-sectional design was used to assess a sample of 260 children aged 7-15 years through a multimodal approach integrating neuropsychological tests (2- and 3-symbol cancellation tasks from the Coimbra Neuropsychological Assessment Battery; Trail Making Test Parts A and B), eye-tracking metrics, and automated facial emotion recognition during rapid naming and emotion recognition tasks. Oculomotor, emotional, and cognitive data were captured simultaneously and in real time, enabling dynamic characterization of cognitive-emotional interactions at millisecond resolution during task performance. A machine learning pipeline based on a random forest classifier, with a gradient boosting surrogate model used for Shapley additive explanations interpretability analyses, analyzed these data to differentiate children with lower attentional performance from children with normative attentional performance.

**Results:**

The random forest classifier achieved the best performance in the exploratory 80/20 hold-out evaluation (area under the receiver operating characteristic curve=0.9527; *F*_2_-score=0.9055; accuracy=94.2%; sensitivity=88.5%; specificity=100%). To validate the absence of test-set selection bias, a nested 5×5 stratified cross-validation was conducted with model selection based exclusively on inner-fold *F*_2_-scores. The negligible difference between nested cross-validated area under the curve (AUC; mean 0.9506, SD 0.0378) and the hold-out AUC (ΔAUC=0.0021) confirmed that the original result was not inflated. Children with lower attentional performance showed increased emotional reactivity, greater gaze instability, fragmented visual exploration patterns, higher variability in fixation behavior, and more frequent impulsive saccadic movements during emotionally salient tasks. Anger and fear stimuli showed stronger discriminative value across attentional performance levels, suggesting that emotional processing modulates attentional allocation.

**Conclusions:**

The simultaneous, real-time capture of eye-tracking, emotion recognition, and neuropsychological data, combined with post hoc analysis within an interpretable machine learning framework, may support the identification of behavioral patterns associated with attentional functioning in children. This multimodal approach provides ecologically grounded and behavior-centered indicators that may support future screening and intervention strategies in educational and clinical contexts.

## Introduction

Attention-deficit/hyperactivity disorder (ADHD) is a neurodevelopmental condition with a global prevalence estimated at approximately 8% in children and adolescents [1], characterized by persistent patterns of inattention, hyperactivity, and impulsivity that interfere with functioning or development [2]. A diagnosis of ADHD requires a full clinical evaluation that cannot be based solely on screening measures. In addition to the core symptoms, impairments are also observed in executive functions (EFs; including working memory, inhibitory control, and cognitive flexibility), as well as emotion dysregulation and attentional processes (sustained/selective attention), which collectively contribute to academic, social, and emotional difficulties [3-5].

The EFs are a set of interrelated higher-order cognitive processes that emerge and develop during early childhood, supporting attention regulation, suppression of distractions, and the planning and coordination of goal-directed tasks [3,5,6]. Key components include working memory (holding and manipulating information), inhibitory control (suppressing impulsive responses), cognitive flexibility (the ability to shift perspectives), and processing speed (the efficiency of cognitive processing). These foundational skills are crucial for many developmental outcomes, including social, emotional, and academic achievement [7,8]. Early childhood represents a sensitive period for the maturation of executive systems, particularly within the prefrontal cortex, which underpins these functions. Deficits in EF during this period are associated with decreased resilience, lower self-regulatory efficacy, and increased behavioral problems in school-age children, and are longitudinally linked to increased risk of neurodevelopmental difficulties [9-11].

A growing body of research highlights the role of EFs in emotion regulation. A comprehensive study found that working memory, inhibitory control, cognitive flexibility, and processing speed play an important role in the regulation of emotional responses and behavioral adaptation [12]. New neurodevelopmental evidence suggests reduced spontaneous activity in frontal regions associated with executive and inhibitory control, while adolescents exhibit more widespread alterations, including cerebellar and sensorimotor areas, suggesting age-dependent differences [13]. Longitudinal evidence further shows that EF and processing speed are closely linked to attentional difficulties, each contributing uniquely to inattentive behaviors [14]. These executive deficits are closely linked to emotional dysregulation, including increased reactivity and difficulties in emotion recognition, and are associated with elevated risk for anxiety and mood difficulties [4,15].

Despite growing recognition of attentional functioning as a multimodal phenomenon, diagnosing ADHD remains challenging, often relying on clinical interviews, behavioral observations, and rating scales that may be influenced by contextual and interpretative biases. As a result, growing attention has been given to data-driven, noninvasive technologies to complement traditional assessments [16]. One such technology is eye-tracking, which has emerged as a promising tool for the objective assessment of attentional mechanisms across cognitive, clinical, and developmental contexts [17,18]. Visual attention is dynamically expressed through eye movements, which reflect how visual information is organized and processed during task performance. Eye movements are therefore not merely motor responses, but functional indicators of attentional allocation, inhibitory control, visual exploration strategies, and visual-cognitive regulation [19,20]. These processes can be objectively examined through eye-movement patterns, including fixation stability, gaze dispersion, saccadic regulation, and visual scanning organization, which provide measurable indicators of attentional control and cognitive efficiency [21]. Children with lower attentional performance have been shown to exhibit atypical gaze patterns, including reduced attention to others’ eyes during emotion recognition tasks [22], as well as broader difficulties in processing emotional scenes [23].

According to Barkley’s [24] theoretical model, ADHD may arise from a primary deficit in behavioral inhibition, disrupting the development of EFs such as working memory, self-directed speech, emotion regulation, and planning. Recent eye-tracking studies have provided further insight into visual attention and oculomotor control in children, advancing understanding of underlying neurocognitive processes [25,26]. However, traditional assessment approaches often fail to capture the dynamic, moment-to-moment interplay between gaze behavior and emotional reactivity that characterizes real-world attentional functioning [27-29]. Addressing these gaps, this study aims to develop a rapid and ecologically valid method for identifying individualized cognitive-emotional-attentional profiles in children and adolescents, grounded in the premise that attentional functioning is inherently linked to emotional and social cognition.

By integrating eye-tracking data with measures of EF and emotional regulation, this study adopts a multimodal neurovisual perspective on attentional functioning, aiming to characterize behavioral profiles associated with differences in visual-attentional regulation, emotional processing, and cognitive control. To this end, the study uses a machine learning (ML) classification approach in which multiple algorithms are used.

The random forest classifier was used as the primary model within a multimodal analytical framework integrating neuropsychological measures, emotion-processing tasks, and eye-tracking indicators to characterize attentional-functioning profiles in children and adolescents. A key methodological feature of this study is the integration of oculomotor, facial-emotional, and cognitive data collected within a single platform, enabling the analysis of multimodal behavioral streams with high temporal precision. The use of ML algorithms, combined with explainability techniques such as Shapley additive explanations (SHAP), may support strong predictive performance while providing interpretable outputs in clinical and educational settings [30]. However, it should be emphasized that the approach used in this study characterizes behavioral patterns of attentional functioning rather than establishing clinical classifications and is therefore limited to a screening process based on psychometric and behavioral indicators, not representing a clinical diagnosis, which requires a comprehensive multidisciplinary assessment by qualified professionals. In this study, behavioral profiles refer to recurring multimodal patterns derived from neuropsychological, oculomotor, and emotional measures associated with different levels of attentional functioning. These profiles are identified through a data-driven approach and do not represent clinical categories or diagnostic classifications; rather, they reflect variability in attentional, emotional, and visual-processing dynamics during task performance.

Building on the previous theoretical and empirical foundations, the following hypotheses were formulated:

- H1: Children with lower attentional performance will exhibit less efficient and more unstable oculomotor patterns compared to children with higher attentional performance, reflected in greater gaze dispersion, increased variability in fixation durations, less organized visual exploration, and a higher frequency of impulsive saccadic movements;
- H2: Emotional stimuli are expected to differentially affect attentional allocation, with children showing lower attentional performance exhibiting distinct gaze patterns and emotional responses compared to children with higher attentional performance.
- H3: Children with lower attentional performance will exhibit weaker integration between gaze behavior and facial emotional expression patterns, reflected in greater variability across both domains.

This study aims to advance understanding of the mechanisms underlying attentional and emotional processing and to inform the development of tailored interventions based on cognitive and emotional profiles. By leveraging behavioral and emotional data, this approach enables faster and more consistent evaluations [31]. By integrating eye-tracking, emotion analysis, and cognitive measures collected in real time, the proposed framework provides a more automated screening model for identifying subtle behavioral patterns through multimodal analysis.

## Methods

### Participants and Procedure

A total of 260 children aged 7-15 (mean 10.36, SD 2.50; median 10, IQR 8-12) years from a public elementary school in the northern region of Portugal participated in the study. Of the total participants, 142 (54.6%) were male. The age distribution was mainly concentrated among children aged 7 years (n=46, 17.7%), 12 years (n=45, 17.3%), and 8 years (n=41, 15.8%), while the remaining participants were distributed across the other age groups within the 7-15-year range. Regarding attentional performance, the sample was evenly distributed, with 130 children classified as having adequate attentional performance (“good”) and 130 classified as having poorer attentional performance (“poor”), each group representing 50% of the sample. A balanced distribution was obtained through a case-control sampling procedure, preserving the ecological validity of both groups while ensuring methodological rigor in the context of ML classification. Sex distribution was also comparable between groups: the good attentional performance group comprised 60 female and 70 male individuals, whereas the lower attentional performance group comprised 58 female and 72 male individuals.

Inclusion criteria comprised children aged 7-15 years attending a regular school setting. Exclusion criteria were based on the student’s school records: (1) not having Portuguese as a first language, (2) inability to recognize the alphabet, (3) presence of any motor impairments that could interfere with task performance, and (4) intellectual disability or developmental delay. Only participants with complete data across all experimental conditions were included in the final sample, ensuring consistency and robustness for subsequent multimodal and ML analyses.

Eye movements were recorded using the Tobii Eye Tracker 4C, a remote, infrared-based binocular eye-tracking system operating at 90 Hz. According to manufacturer specifications, the system provides spatial accuracy of approximately 0.4°-0.5° and a precision of less than 0.2° root mean square under optimal calibration conditions.

All assessments were conducted under standardized environmental conditions, including controlled ambient lighting and a fixed viewing distance of approximately 60 cm between the participant and the display monitor. Participants were seated comfortably, and a standardized 9-point calibration procedure was performed individually prior to each assessment session. Recalibration was undertaken whenever tracking quality was deemed suboptimal. Only recordings demonstrating stable binocular tracking and complete data acquisition across all experimental conditions were retained for subsequent analysis.

Experimental tasks were administered under fixed presentation parameters within the Neurobiosensorial platform, which integrates real-time eye-tracking with automated facial emotion recognition during rapid naming and emotional face recognition paradigms. Facial emotional responses were recorded via webcam and analyzed using the HSE-AffectNet framework embedded in the platform. The system was designed to detect basic facial emotions (happiness, sadness, anger, fear, and surprise), which are widely regarded as universal expressions within Ekman’s theoretical framework and are comparatively less dependent on linguistic processing.

The assessment protocol was administered individually by a trained psychologist during a single session lasting approximately 45-60 minutes, in the absence of caregivers. Although no formal breaks were scheduled, task demands were systematically alternated to mitigate fatigue and sustain participant engagement. The protocol was previously piloted to ensure developmental appropriateness for the target age group, thereby supporting standardized data acquisition while minimizing potential sources of external interference.

The protocol followed a fixed sequence: (1) 2- and 3-symbol cancellation tasks from the Coimbra Neuropsychological Assessment Battery (CNAB), (2) Trail Making Test (TMT), Parts A and B, and (3) eye-tracking–based tasks, including Rapid Naming of Shapes and Colors and emotion recognition. In line with the study’s hypotheses, oculomotor indices derived from eye-tracking (eg, fixation variability, gaze dispersion, and saccadic patterns) were used to capture attentional stability and visual efficiency, while emotion-related measures were used to examine the modulation of attention by emotionally salient stimuli.

Cutoff scores from assessment instruments were used to support screening-based interpretations of attentional difficulties, applied solely for research purposes and not for clinical diagnosis. This distinction is critical given the study’s focus on characterizing variability in attentional functioning rather than establishing diagnostic categories.

### Ethical Considerations

The study was conducted in accordance with the Declaration of Helsinki and was approved by the Clinical Review Board, the Ministry of Education (approval number 0786500001), and the school principal. Parental or legal guardian written consent was obtained for all participants, authorizing the use of the children’s data for research purposes. Participation was voluntary with no compensation, and confidentiality and the right to withdraw at any stage were guaranteed. All data were anonymized and deidentified prior to analysis, and appropriate data protection measures were implemented to safeguard participants’ information.

### Assessment Instruments

#### Sociodemographic Questionnaire

This questionnaire was specifically designed for the purpose of this study to characterize the sample (eg, age, gender, educational level, and parental occupational status).

#### Neuropsychological Assessment

##### The Cancellation Scale From the CNAB

This subscale evaluated attention and EFs in children [32]. The task required the child to visually scan a sheet filled with various symbols and mark specific target stimuli within a specified time limit. Performance was measured based on the number of correctly identified targets and the number of commission and omission errors. Higher accuracy and fewer errors indicate better attentional control and executive functioning.

In the original validation studies, the CNAB Cancellation subtests showed adequate psychometric properties for the pediatric population, including satisfactory internal consistency (Cronbach α values ranging from 0.78 to 0.86 across age groups) and good interrater reliability (intraclass correlation coefficients >0.90). Construct validity was supported by significant correlations with other established measures of attention and processing speed, and discriminant validity was evidenced by the subtest’s ability to differentiate between typically developing children and those with attention-related disorders [32]. In this study, only the 2- and 3-symbol cancellation tasks from the CNAB were used, each consisting of a single trial; therefore, internal consistency indices such as Cronbach α could not be calculated. Evidence of reliability for the current application relied on prior validation studies of the CNAB, which have reported acceptable stability and sensitivity coefficients for these tasks in similar populations [32]. A screening cutoff score of 7 or below was also used as an indicator of potential attentional difficulties [32].

##### The TMT (CNAB Subscale)

This subscale [32] assessed attention and EFs in children and consisted of 2 parts: Trail A and Trail B. In Trail A, the child is asked to draw lines connecting numbers in ascending order (eg, 1-2-3...), while in Trail B (applied to children aged 7 years or older, as it requires knowledge of the alphabet), the task becomes more complex by requiring the child to alternate between numbers and letters in ascending order (eg, 1-A-2-B...). Time to complete the instrument is measured in seconds. Errors are not directly scored, but they can influence overall performance, as the examiner intervenes when a mistake occurs by pointing out the last correct response, thereby increasing completion time. Each part of the Trail Task consists of a single trial; thus, internal consistency indices could not be computed. Moreover, in the absence of a test-retest design, it was not possible to directly assess temporal stability. Reliability evidence for the present application is therefore based on previously published psychometric studies of the TMT, which have shown satisfactory stability and validity in similar populations [32]. In this study, performance was interpreted using established screening cutoff scores, with values <7 indicating poorer attention performance [32].

##### The Rapid Automatized Naming of Shapes and Colors (CNAB Subscale)

This instrument [32] was integrated with eye-tracking and facial emotion recognition tools. The child was presented with a visual sheet containing a sequence of shapes (triangles, circles, and squares) in different colors (yellow, green, and blue) and was asked to name each shape and its corresponding color line by line, as quickly and accurately as possible. During task performance, an eye-tracker recorded oculomotor activity to analyze visual exploration patterns, including fixation count and duration, regressions, and loss of sequence. Raw gaze data were processed using a velocity-based fixation classification algorithm (Identification by Velocity Threshold) with a minimum fixation duration of 100 ms. Additional metrics included saccadic amplitude, interfixation interval variability, and the coefficient of variation of fixation duration. These measures index attentional stability, visual exploration efficiency, and visual-cognitive organization. Composite indicators of visual efficiency and attentional instability were derived from gaze dispersion, fixation variability, and saccadic transition patterns. Simultaneously, a facial emotion recognition system records spontaneously displayed emotions, providing insight into the affective experience associated with cognitive performance. This combined approach may support a multimodal examination of attentional and emotional processes during rapid visual-verbal tasks. No screening cutoff score was used; instead, comparative analyses were based on the distribution of total response times across participants. Children were divided into groups representing higher and lower task performance, determined by e-based segmentation of total naming time (with the lowest quartile representing faster, more efficient performance and the highest quartile indicating slower processing). Between-group comparisons were then conducted to examine differences in accuracy, eye-tracking parameters (eg, fixation count, fixation duration, and regressions), and patterns of emotional expression.

#### Emotional Processing Multimodal Assessment

##### Emotion Recognition

This task was performed by the Neurobiosensorial program [33]. This platform was specifically designed to enable the simultaneous, real-time recording of eye movements and facial emotional responses within a single integrated environment. This program tracks eye movements and pinpoints eye position while executing a specific task (emotion naming), while detecting emotional responses [33]. Eye-tracking measures eye movement at millisecond intervals, providing insight into the cognitive processes involved in task interpretation. The participant’s facial expressions are analyzed using HSE-AffectNet and webcam image analysis combined with AI technology to identify emotions experienced throughout the task [34]. The program’s interface relies on 27 simple facial recognition points (eg, pupils, eyebrows, and the nose area) as reference markers. During each task, whether it involves a higher cognitive demand or emotional identification, a list of the emotions felt by the child throughout the experience is generated as an outcome [34].

##### Emotion Naming

This task was based on the emotion recognition paradigm developed by Paul Ekman, adapted to assess children’s ability to identify basic facial emotions [35]. The task focuses on 5 fundamental emotions: happiness, sadness, anger, surprise, and fear. In each trial, the child is presented with 4 images of facial expressions, each depicting a distinct emotion. The child is then asked to identify which images correspond to the target emotion verbally indicated by the examiner (eg, “Which face shows sadness?”). The primary variables are the ocular movement patterns and the emotions experienced by the participants, recorded simultaneously and in real time using an eye-tracking system and a camera-based facial emotion recognition system. This synchronized, dual-stream capture allows direct examination of moment-to-moment interactions between where a child looks and what emotional state they express. While the child is asked to identify the target emotion among the 4 images, the system captures their visual scanning paths, fixation points, and saccades, providing insight into the attentional strategies used during emotion recognition. Raw gaze data were processed using a velocity-based fixation classification algorithm (Identification by Velocity Threshold), with a minimum fixation duration threshold of 100 ms [34]. This procedure segmented gaze data into fixations and saccades based on movement velocity profiles.

Areas of interest (AOIs) were defined based on the spatial structure of each task. In the rapid automatized naming task, AOIs corresponded to the stimulus grid and sequential naming regions [36]. In the emotion recognition task, AOIs were defined around facial stimuli, including key regions such as the eyes, mouth, and overall face, given their relevance for social and emotional processing. A tolerance margin was applied to account for minor tracking variability [36].

Composite indicators of attentional instability and visual efficiency were derived from gaze dispersion, fixation variability, and saccadic transition patterns, capturing higher-order interactions between visual attention, emotional reactivity, and oculomotor regulation. Eye-tracking data were analyzed using custom computational pipelines. Extracted metrics included fixation count (total fixations within task-relevant AOIs), mean fixation duration (attentional engagement), saccadic amplitude (visual exploration dynamics), and gaze dispersion (spatial distribution of attention) [35,36].

Additionally, the facial emotion recognition system recorded the spontaneous emotional reactions elicited in the child while observing the stimuli, assessing the child’s ability to recognize emotions and their affective responses, as well as the visual processing behaviors associated with each emotion. Some participants may spend more time on the eye region, while others may focus on the mouth or the overall facial contour. Additionally, the number and sequence of oculomotor metrics can differ depending on attentional control, processing speed, and familiarity with emotional expressions. This natural variability affects the consistency of the data when aggregated across participants; however, it remains meaningful as it reflects genuine differences in attentional and cognitive processing styles [34,36].

### Data Analysis

#### Overview

Two significant sources were used: (1) clinical test results (Cancellation, Trail A, and Trail B) providing screening-based attentional-performance labels (1=lower attentional performance, 0=typical attentional performance) and (2) behavioral metrics collected from eye-tracking and emotion recognition during 6 tasks: “Rapid Naming of Shapes and Colors-NRFC),” “anger,” “joy,” “sadness,” “fear,” and “surprise.” A child was included only if all metrics were available across all tasks.

A total of 30 variables selected from an initial set of 61 engineered features were used in the final model. Each variable reflects cognitive-emotional interaction patterns. The features were selected spanning 5 behavioral domains: (1) oculomotor and attentional metrics derived from eye-tracking (eg, gaze path length, dispersion, fixation duration variability, interfixation interval variability, and visual efficiency), (2) emotion-specific expression and gaze features (eg, expression duration, dwell time, and gaze allocation per emotional category), (3) aggregated emotional indices (eg, emotional intensity, transition rate, valence balance, emotion bias duration, and number of active emotions), (4) composite cognitive-emotional indices (Attentional Instability Index, Gaze-Emotion Congruence Index, Emotional Reactivity Index, Visual Efficiency Index), and (5) a demographic covariate (age). These indices represent multimodal neurobehavioral markers integrating visual-attentional, emotional, and oculomotor dimensions.

#### Preprocessing

The raw integrated dataset contained variables for each child, representing both aggregated and emotion-specific behavioral metrics. No missing values were present in the final preprocessed dataset. The final dataset comprised 260 participants, with a balanced distribution across groups (130 with attentional difficulties; 130 with typical performance). The data pipeline followed a structured three-step procedure prior to model training:

- Step 1: data cleaning and preprocessing. Categorical variables were encoded using one-hot encoding. Missing values in fixation-related metrics were encoded with zeros to ensure dataset completeness and maintain consistency across participants. During this step, absent behavioral events, such as fixations in a stimulus zone not visited by a given participant, were assigned a value of zero, reflecting the absence of that behavior rather than a measurement gap. This domain-appropriate substitution resulted in a complete feature matrix with no missing values at the modeling stage. This preprocessing stage produced a clean, analysis-ready dataset.
- Step 2: feature engineering. Behavioral metrics derived from eye-tracking and emotion recognition were aggregated at the participant level using summary statistics (mean and SD) across trials. In addition, composite indices were computed to capture higher-order cognitive-emotional processes, including the Attentional Instability Index, Oculomotor Control Index, Emotional Reactivity Index, and Visual Efficiency Index). This process resulted in an initial feature set of 61 variables.
- Step 3: feature selection. Feature selection was conducted using mutual information scoring combined with redundancy filtering (correlation threshold |r|<0.80). This procedure yielded a final set of 30 features spanning oculomotor attention metrics, emotion-specific gaze and expression variables, aggregated emotional indices, composite cognitive-emotional indices, and age. All feature selection procedures were performed exclusively on the training set to prevent information leakage.

The final dataset was partitioned into training (n=208, 80%) and testing (n=52, 20%) subsets using a stratified split (random state=42) to preserve class proportions. The held-out test set was strictly excluded from preprocessing, fitting, feature selection, and hyperparameter optimization procedures.

#### ML Model Definition

The classification model was based on 7 classification architectures that were trained and compared, including 5 individual learners—gradient boosting, extreme gradient boosting (XGBoost), light gradient boosting machine (LightGBM), random forest, and support vector machine (SVM)—as well as 2 ensemble strategies: a soft-voting ensemble combining the top 3 models based on cross-validated area under the receiver operating characteristic curve (AUC-ROC; random forest, XGBoost, and gradient boosting), and a stacking ensemble using a logistic regression meta-learner trained on out-of-fold predictions from all base models.

Hyperparameters were independently optimized using Optuna (Bayesian optimization, tree-structured Parzen estimator, 15 trials per model), evaluated via 5-fold stratified cross-validation AUC-ROC on the training set. Optimized hyperparameters and corresponding AUC-ROC scores are reported in Table 1. A 5×3 repeated stratified K-fold was additionally applied to the training data to assess hyperparameter stability; the unbiased performance estimate is provided by the nested 5×5 cross-validation described below.

**Table 1:** Models trained, hyperparameters, and Optuna cross-validated area under the curve (AUC-CV).

Model	Type	Key hyperparameters (Optuna best)	AUC-CV	*F*_2_-score (test)
Gradient boosting	Tree ensemble	n_estimators=102, max_depth=6, lr=0.111, subsample=0.865, min_samples_leaf=16	0.9635	0.9055
XGBoost^a^	Boosting	n_estimators=695, max_depth=8, lr=0.158, subsample=0.708, colsample=0.996	0.9566	0.8661
LightGBM^b^	Boosting	n_estimators=768, max_depth=8, lr=0.023, subsample=0.509, colsample=0.739	0.9614	0.8984
Random forest^c^	Bagging	n_estimators=539, max_depth=15, min_samples_leaf=2	0.9528	0.9055
SVM^d^	Kernel	kernel=rbf, C=2.971, gamma=0.039	0.8633	0.8214
Voting ensemble	Soft voting	Top-3 models by AUC^e^ (RF^f^ + XGB^g^ + GBM^h^)	0.9589	0.9055
Stacking ensemble	Stacking	Base: top-5 models; Meta: logistic regression (C=1.0)	0.9596	0.9055

^a^XGBoost: extreme gradient boosting.

^b^LightGBM: light gradient boosting machine*.*

^c^Random forest selected as primary screening framework/predictive model.

^d^SVM: support vector machine.

^e^AUC: area under the curve.

^f^RF: random forest.

^g^XGB: extreme gradient boosting*.*

^h^GBM: gradient boosting machine.

Model generalization was estimated using a 5×3 repeated stratified K-fold cross-validation strategy applied exclusively to the training set (n=208). This procedure was used to estimate hyperparameter stability during model development. Repeating the 5-fold procedure 3 times (15 folds total) reduces the variance of the performance estimate and provides a more robust basis for comparing models during training.

Although random forest did not achieve the highest cross-validated area under the curve (AUC-CV; AUC-CV=0.9528; full rankings in Table 1), it was selected as the primary model based on its *F*_2_-score (0.9055) and zero false positives (FPs) in the exploratory 80/20 hold-out evaluation (Table 2). The absence of test-set inflation was confirmed by nested 5×5 cross-validation (area under the curve [AUC]: mean 0.9506, SD 0.0378; ΔAUC: mean 0.0021, SD 0.0378).

**Table 2:** Classification performance—exploratory 80/20 hold-out.

Model	AUC-ROC^a^ (test)	AUC-CV^b^ (train, 5×3), mean (SD)	Accuracy	Precision	Recall (sensitivity)	Specificity	*F*_1_-score	*F*_2_-score	FP^c^	FN^d^
Random forest^e^	0.95	0.95 (0.03)	0.94	1.0000	0.8846	1.00	0.94	0.91	0	3
Gradient boosting	0.94	0.96 (0.02)	0.94	1.0000	0.8846	1.00	0.94	0.91	0	3
Voting ensemble	0.95	0.96 (0.03)	0.94	1.0000	0.8846	1.00	0.94	0.91	0	3
Stacking ensemble	0.95	0.96 (0.03)	0.94	1.0000	0.8846	1.00	0.94	0.91	0	3
LightGBM^f^	0.94	0.96 (0.03)	0.92	0.9583	0.8846	0.96	0.92	0.90	1	3
XGBoost^g^	0.95	0.96 (0.03)	0.90	0.9565	0.8462	0.96	0.90	0.87	1	4
SVM^h^	0.81	0.86 (0.05)	0.69	0.6389	0.8846	0.50	0.74	0.82	13	3

^a^AUC-ROC: area under the receiver operating characteristic curve.

^b^AUC-CV: cross-validated area under the curve.

^c^FP: false positive.

^d^FN: false negative.

^e^The primary clinical model uses an optimized classification threshold of 0.48.

^f^LightGBM: light gradient boosting machine.

^g^XGBoost: extreme gradient boosting.

^h^SVM: support vector machine.

The random forest classifier was selected as the primary screening framework based on its *F*_2_-score (0.9055) and zero FPs on the held-out test set. The *F*_2_-score weights recall twice as much as precision, prioritizing clinical sensitivity; the cost of a missed lower-attentional-performance case (false negative [FN]) substantially exceeds that of an unnecessary referral (FP). Although gradient boosting achieved the highest cross-validated AUC, the random forest demonstrated comparable discrimination while providing a more favorable balance between sensitivity and specificity for screening. Its robustness was further confirmed through nested 5×5 cross-validation, achieving a mean AUC-ROC of 0.9506 (SD 0.0378), *F*_2_-score of 0.8953 (SD 0.0664), recall (sensitivity) of 0.8923 (SD 0.0877), specificity of 0.9154 (SD 0.0740), and accuracy of 0.9038 (SD 0.0408). The minimal difference between the nested cross-validation AUC and the held-out test AUC (ΔAUC=0.0021, smaller than the cross-validation SD) indicates that model performance was stable and showed no evidence of performance inflation, supporting its selection as the final screening framework. LightGBM (AUC-CV=0.9614) produced one FP and a lower *F*_2_-score (0.8984) on the test set, making it suboptimal for clinical screening where minimizing false referrals is a priority. Among all models with FP=0 and *F*_2_-score=0.9055 (gradient boosting, voting ensemble, stacking ensemble, and random forest), the random forest achieved the highest test-set AUC-ROC. A gradient boosting model with identical features and data split was trained as a surrogate solely for SHAP interpretability analysis, as TreeExplainer requires direct access to the underlying tree structure.

#### Training and Validation Strategy

The held-out test set was not used during preprocessing, feature selection, or hyperparameter optimization. In the exploratory phase, model selection was informed by *F*_2_-score and the false-positive rate observed on the hold-out set. The absence of test-set inflation was subsequently confirmed by nested 5×5 cross-validation (ΔAUC: mean 0.0021, SD 0.0378).

Each model was fitted on the full training set (n=208) using its Optuna-optimized hyperparameters. Final evaluation was performed once on the held-out test set (n=52, 26 per class) using the decision threshold optimized on the training cross-validation folds. Performance was assessed using AUC-ROC (threshold-independent), accuracy, precision, recall (sensitivity), specificity, *F*_1_-score, and *F*_2_-score. Recall was designated as the clinically primary metric, with *F*_2_-score used for model selection.

In addition, a threshold sensitivity analysis was conducted across 9 decision thresholds (0.25 to 0.65 in steps of 0.05) for the primary model, to characterize the precision-recall trade-off and support clinical deployment decisions for different use cases (mass screening vs diagnostic confirmation).

## Results

### Classification Performance

Among the 7 candidate models, random forest, gradient boosting, voting ensemble, and stacking ensemble all achieved *F*_2_-score=0.9055 with 0 FPs; random forest was selected for its highest AUC-ROC within this group. On the held-out test set (n=52), the random forest classifier (threshold=0.48, Optuna-optimized) achieved an AUC-ROC of 0.9527, accuracy of 94.23%, precision of 100%, recall of 88.46%, and *F*_2_-score of 0.9055 (Table 2). Every child with typical attentional performance was correctly excluded (FP=0); 3 children with lower attentional performance were missed (FN=3, FN rate=11.54%). Nested 5×5 cross-validation confirmed the absence of test-set inflation (AUC: mean 0.9506, SD 0.0378; ΔAUC=0.0021), supporting the validity of the reported estimates.

### Sensitivity Analysis

To assess whether the facial expression confidence score reflected a genuine behavioral signal or acquisition-quality effects, a sensitivity analysis was conducted comparing the full model against a reduced version excluding this feature, with all preprocessing, hyperparameter optimization, and evaluation procedures kept identical. Classification performance remained essentially unchanged (ΔAUC=0.000; Δrecall=0.000; n=52), and SHAP-based feature rankings showed high stability (Spearman ρ=0.959; *P*<.001; 8/10 top features identical), indicating that the model’s predictive behavior does not depend on this feature and is unlikely to be driven by acquisition-quality differences. Full results are presented in Table S1 in Multimedia Appendix 1 and Table 3.

**Table 3:** Sensitivity analysis: facial expression confidence score.

Metric	Full model (30 features)	Reduced model (29 features)	Δ^a^
AUC-ROC^b^	0.9527	0.9527	+0.0000
Recall (sensitivity)	0.9231	0.9231	+0.0000
*F*_2_-score	0.8955	0.9023	–0.007
SHAP^c^ rank correlation (ρ)	—^d^	0.959^e^	—

^a^Δ = full model – reduced model.

^b^AUC-ROC: area under the receiver operating characteristic curve.

^c^SHAP: Shapley additive explanations.

^d^Not available.

^e^*P*<.001.

### Model Comparison

All 7 models were evaluated on the same held-out test set (Table 2). LightGBM and XGBoost produced FP=1 on the test set and were therefore excluded from the primary model selection. Within the FP=0 group, random forest achieved the highest AUC-ROC and was retained as the primary model. The SVM substantially underperformed (AUC-CV=0.8633; test AUC=0.8092; FP=13) and was excluded from further consideration. A gradient boosting surrogate was used for SHAP-based interpretability analysis.

### Clinical Threshold Analysis

A threshold sensitivity analysis was performed for the gradient boosting surrogate across 9 decision thresholds (0.25-0.65). Performance remained stable between 0.25 and 0.55 (recall=88%; specificity=96%; FP=1; FN=3), reflecting strong probabilistic separation. Above threshold 0.60, recall drops to 84.6% (FN=4); this range is therefore not recommended. The primary random forest model operates at the Optuna-optimized threshold of 0.48, achieving FP=0 and specificity of 100% (full results in Table 2).

### Feature Importance and SHAP Analysis

Feature importance was assessed using SHAP [30] values computed via Tree Explainer on a gradient boosting surrogate model trained on the same 30 features, the same stratified 80/20 split, and the same preprocessing pipeline as the primary random forest classifier (surrogate AUC-ROC=0.9438). This surrogate-based approach was adopted because Tree Explainer yields exact Shapley values for gradient boosting’s additive ensemble of shallow trees, whereas application to a random forest requires approximations that degrade under correlated predictors [37,38]. This strategy is consistent with established practice in clinical ML interpretability research [38,39].

Surrogate fidelity was confirmed by a Spearman rank correlation of ρ=0.78 (*P*<.001) between GBM-SHAP and random forest Gini-impurity rankings, with the 4 most influential features shared across both: attentional bias toward emotional stimuli, visual attention toward joyful facial expressions, visual exploration efficiency, and the proportion of time expressing sadness-related facial expressions. This consistency ensures that the clinical narrative derived from SHAP faithfully reflects the primary model’s discriminative logic. It should be noted that the surrogate exhibited FP=1 across all tested thresholds, reflecting stochastic variability inherent to independent model fitting; this does not affect the validity of the SHAP analysis, as both models achieved comparable AUC-ROC (ρ=0.78). SHAP contributions were aggregated by feature group and by facial emotion stimulus category.

Aggregating SHAP contributions by feature category reveals the relative importance of each behavioral domain to the classification decision, as represented in Table 4.

**Table 4:** Shapley additive explanations (SHAP) weight distribution by feature group.

Feature group	Total absolute SHAP value (%)
Per-emotion features (expression + gaze)	3.6677 (37.1)
Eye-tracking (oculomotor/attention)	3.2362 (32.7)
Aggregated emotion	2.4375 (24.6)
Composite indices (VEI^a^/AII^b^/OCI^c^/ERI^d^/SEI^e^)	0.3895 (3.9)
Demographic (age)	0.1641 (1.7)

^a^VEI: Visual Efficiency Index.

^b^AII: Attentional Instability Index.

^c^OCI: Oculomotor Control Index.

^d^ERI: Emotional Reactivity Index.

^e^SEI: Social Exploration Index.

The 3 most informative domains collectively account for 94.4% of total SHAP weight. Per-emotion features (37.1%) capture how children’s facial expressions and gaze allocations differ per emotional stimulus, particularly fear (|SHAP|=0.753), sadness (|SHAP|=0.717), and anger expressions (|SHAP|=0.571). Eye-tracking metrics (32.7%) capture oculomotor instability and scanning inefficiency: higher gaze path length, greater fixation duration variability, and elevated interfixation interval variability are all associated with the lower attentional-performance profile. Aggregated emotion indices (24.6%) are dominated by the mean of recognition bias (|SHAP|=1.726), the ratio of fixations toward negative vs positive emotional faces, which is the single most predictive feature in the model.

The results indicate that lower attentional performance prediction derives from converging evidence across emotional face processing, oculomotor control, and cross-emotion behavioral consistency, consistent with theoretical accounts linking attentional difficulties in childhood to lower attentional performance in both executive attention and socioemotional processing (Table 4).

Among the 30 selected features, the subset of per-emotion features, derived specifically from 1 of the 5 emotional face stimuli (happiness, fear, sadness, anger, and surprise), accounts for a cumulative SHAP weight of 3.667, representing 37.1% of the total model importance. The contributions are markedly unequal across emotional categories:

- Happiness (SHAP=1.338, 36.5%): the dominant emotional stimulus. The majority of this effect was driven by the average visual attention directed toward joyful facial expressions (SHAP=1.249), the proportion of fixations directed toward the joyful face. Children with lower attentional performance show an atypical allocation of visual attention to positive emotional faces, a pattern consistent with altered social orienting.
- Fear (SHAP=0.861, 23.5%): the second most informative stimulus, combining sustained fear expression duration (SHAP=0.753) and its variability (SHAP=0.108). Prolonged and variable fear expressions in the lower attentional performance group suggest heightened anxious reactivity and difficulty recovering from negative emotional arousal.
- Sadness (SHAP=0.717, 19.5%): captured entirely by the proportion of time spent expressing sadness-related facial expressions. Elevated sadness expression was associated with the lower attentional-performance profile, potentially reflecting lower task motivation and negative affective responses during sustained attention demands.
- Anger (SHAP=0.703, 19.2%): contributed through expression duration (SHAP=0.571) and gaze allocation toward the angry face (SHAP=0.132). Together, these indicate both prolonged angry expression episodes and an attentional bias toward threat stimuli, a pattern associated with emotional dysregulation in children with lower attentional performance.
- Surprise (SHAP=0.027, 0.7%): the least informative emotional stimulus. The short duration of expressed surprise provides minimal discriminative power, suggesting that surprise stimuli do not elicit substantially differentiated behavioral responses between the 2 groups.

The 3 negative emotional stimuli combined (fear, sadness, and anger) account for 62.2% of the emotional stimulus contribution, underscoring the disproportionate role of negative affect processing in the attentional-functioning classification. Happiness, while positive, ranks first partly because gaze allocation toward a joyful face is a sensitive marker of social attention, a process known to be altered in children with attentional and executive difficulties.

### Multimodal Behavioral Profiles Associated With Attentional Functioning

#### Overview

To move beyond aggregate performance metrics and toward a clinically meaningful characterization of the model’s discriminative logic, 2 composite behavioral profiles were constructed. These profiles describe the patterns of oculomotor, attentional, and facial-emotional behavior that systematically differentiate children at high risk of lower attentional performance from those presenting typical attentional development, as identified through SHAP analysis of the gradient boosting surrogate model.

Each dimension reported below is grounded in the feature’s mean absolute SHAP value, its contribution to the multivariate classification decision, and independent univariate group comparisons (independent-samples *t* tests, n=130 per group, 2-tailed), as well as Cohen *d* effect sizes.

#### Profile A: Neurobehavioral Profile Associated With Lower Attentional Performance

Children with lower attentional performance exhibited a coherent multidimensional behavioral profile characterized by:

- Attentional bias toward negative stimuli and increased gaze allocation to socially salient faces.
- Instability and inefficiency in oculomotor control, reflecting disrupted attentional regulation.
- Diffuse and inflexible emotional expression patterns, combining broader affective range with reduced intensity and slower transitions.

This lower attentional-performance profile emerges from the convergence of these 3 behavioral signatures: a systematic attentional bias toward emotionally salient and threatening stimuli, markedly inconsistent and inefficient oculomotor control, and a pattern of diffuse, emotionally inflexible facial expression. Importantly, no single feature is sufficient to define this profile; rather, it is the combined expression of these dimensions that characterizes the lower attentional-functioning profile.

#### Profile B: Neurobehavioral Profile Associated With Higher Attentional Performance

Children with higher attentional performance exhibited a distinct and active behavioral profile, rather than simply the absence of high-risk markers. This profile reflects efficient, balanced, and flexible cognitive-emotional processing, characterized by adaptive attention allocation, stable and economical oculomotor control, and clear, dynamic emotional expression. Importantly, the low-risk profile emerges from the convergence of three complementary behavioral signatures: balanced attentional deployment across emotional stimuli, consistent and efficient gaze dynamics, and emotionally expressive yet flexible affect regulation. These characteristics represent positive indicators of attentional health rather than merely the inversion of lower-performing attentional features.

Children in the low-risk group, therefore, displayed a coherent and adaptive behavioral profile characterized by:

- Balanced attentional allocation, avoiding overfocus on emotionally salient or threatening stimuli.
- Stable and efficient oculomotor behavior, reflecting organized and sustained attentional control.
- Clear, expressive, and emotionally flexible responses, indicating effective affect regulation.

## Discussion

### Principal Findings

This study aimed to identify multimodal behavioral patterns associated with different levels of attentional functioning, integrating neuropsychological, oculomotor, and emotional measures within an ML framework. Consistent with previous eye-tracking studies, which have demonstrated that objective measures of visual attention can capture subtle attentional impairments in children with lower attentional performance beyond traditional behavioral observation [40,41], the current findings suggest that combining these objective markers may enhance the sensitivity of assessment frameworks. Overall, the results supported the proposed hypotheses, though with some nuances in the strength and specificity of the observed effects.

The results indicate that children with lower attentional performance exhibit less efficient, more unstable oculomotor patterns, characterized by increased gaze instability, greater variability in fixation duration, and more fragmented visual exploration, along with a higher frequency of impulsive saccadic movements. These results are consistent with previous literature indicating that lower attentional performance is associated with impaired oculomotor control and reduced visual efficiency [20,22]. From a neurodevelopmental perspective, such patterns may reflect underlying inefficiencies in EFs, particularly inhibitory control and processing speed, which are critical for regulating visual attention and goal-directed behavior [3,6]. This interpretation aligns with theoretical models suggesting that deficits in behavioral inhibition disrupt attentional regulation [24]. These findings are consistent with H1, supporting the expectation that lower attentional performance is associated with less efficient and more unstable oculomotor behavior.

A distinctive methodological contribution of this study is the simultaneous, real-time capture of oculomotor, emotional, and cognitive-behavioral streams within a single platform. Unlike traditional assessments, in which gaze data, emotional responses, and neuropsychological performance are collected separately and analyzed post hoc, the present approach enables direct observation of dynamic cognitive-emotional interactions as they unfold during task performance. This revealed patterns, such as the cooccurrence of gaze instability and heightened expression of fear or anger within the same task. This approach allows us to identify not only whether a child performs poorly, but also precisely when and in what emotional context attentional dysregulation emerges, providing a richer basis for generating hypotheses about future personalized intervention strategies.

Additionally, emotional stimulus was found to play a significant role in modulating attention allocation. Conditions involving anger and fear showed strong discriminative value between groups, suggesting that attentional processes are particularly sensitive to emotionally salient information. Children with lower attentional performance demonstrated altered gaze patterns and heightened emotional reactivity to these stimuli, suggesting that emotional processing may interfere with attentional control.

The findings are consistent with previous research indicating that lower attentional performance is associated with atypical processing of emotional cues, including altered gaze allocation to socially relevant facial features and difficulties in social-emotional perception [27,42,43]. Furthermore, eye-tracking metrics revealed increased fixation pattern variability and altered saccadic latencies, consistent with difficulties in maintaining goal-directed visual attention and in efficient attentional control. Previous studies have shown that such oculomotor alterations are characteristic of children with ADHD and other neurodevelopmental conditions [22,44].

From a broader perspective, the attentional inefficiencies may contribute to cognitive overload and emotional dysregulation, underscoring the close interaction between attentional and affective processes during development. Children with normative attentional performance, by contrast, exhibited more stable fixation patterns, consistent saccadic control, and lower variability across oculomotor indices. These findings are in line with previous eye-tracking studies indicating that normative developmental profiles are characterized by efficient visual attention allocation and a preference for socially relevant stimuli [40,45]. In this context, the results support H2, indicating that emotional stimuli differentially modulate attentional allocation as a function of attentional performance level. The correspondence between our results and the existing literature supports the use of multimodal behavioral markers as promising behavioral indicators of attentional functioning, providing a reference framework against which deviations related to attentional impairments and emotion-related processes can be identified.

Furthermore, the interaction between gaze behavior and emotional expression emerged as a relevant factor in distinguishing attentional profiles. Composite cognitive-emotional indices, such as the Gaze-Emotion Congruence Index and Emotional Reactivity Index, were identified as highly informative features, highlighting the importance of integrated processing across domains. Children with lower attentional performance showed greater variability in both gaze and emotional expression, suggesting less consistent and less coordinated responses. These findings align with theoretical perspectives that emphasize the interdependence of cognitive control and emotional regulation systems during development [42,46]. Rather than reflecting a complete dissociation between domains, the results point to a pattern of dysregulated integration, in which attentional and emotional processes interact in a less stable and less adaptive manner. Similarly, fear-related stimuli elicited attentional disruptions, including prolonged fixations and difficulty disengaging from threatening cues. These patterns are consistent with eye-tracking findings indicating that children exhibiting attentional difficulties display atypical gaze allocation toward fearful faces, accompanied by reduced accuracy in recognizing threat-related emotions [47]. Neurodevelopmental research suggests that atypical amygdala-prefrontal functional dynamics contribute to these effects, reducing top-down modulation and promoting hypervigilance to threat cues [48,49]. Therefore, the findings support H3, suggesting weaker integration between gaze behavior and emotional expression in children with lower attentional performance.

The integration of multimodal behavioral data into a multimodal ML framework suggests potential applications for early screening and for informing future personalized intervention approaches. SHAP analyses allowed identification of the features that most strongly influenced model predictions, offering concrete targets for interventions to improve gaze stability, visuomotor control, and emotion recognition [41,42]. Importantly, it highlights the dynamic interplay between attention, cognition, and emotion, emphasizing the need for integrated intervention strategies that target multiple domains simultaneously.

The study’s findings support H1, H2, and H3, highlighting a pattern of oculomotor inefficiency, altered emotional modulation of attention, and reduced integration between cognitive and affective processes in children with lower attentional performance. Nevertheless, it is important to note that these findings reflect behavioral patterns associated with attentional functioning and do not constitute clinical diagnoses.

Finally, this study contributes to the growing body of literature advocating ecologically valid and data-driven approaches to the assessment of neurodevelopmental conditions. By integrating oculomotor behavior, emotional processing, and executive functioning, the present results support a more comprehensive understanding of attentional difficulties during development and highlight the value of multimodal assessment models for early identification and intervention.

### Limitations

Despite the promising findings, several limitations warrant consideration. The study was conducted with a relatively small sample size (N=260), which may limit the generalizability of the results and increase the risk of model overfitting, despite nested cross-validation confirming the absence of test-set inflation (ΔAUC=0.0021). The class labels were derived from screening-based cutoff scores (≤7 in the Cancellation task, TMT A, and TMT B) rather than a formal clinical diagnostic gold standard. One feature related to facial expression detection confidence emerged as an influential predictor and showed significant differences between groups. A sensitivity analysis conducted with and without this feature demonstrated that classification performance remained unchanged (ΔAUC=0.000 and Δrecall=0.000) and SHAP-based feature rankings were highly stable (Spearman ρ=0.959; *P*<.001; 8/10 top features identical), indicating that the model’s predictive behavior does not depend on this feature. Nevertheless, a contribution from recording-quality factors (eg, occlusion, lighting, or camera angle) cannot be completely excluded. Consequently, the proposed model should be interpreted as a decision-support or screening tool rather than a definitive diagnostic system. Although the model demonstrated strong internal validation performance through stratified cross-validation and holdout testing, external validation across independent cohorts, larger populations, and multicenter clinical settings remains necessary to further establish its robustness, reproducibility, and real-world clinical applicability. The protocol included tasks that were conducted in semicontrolled environments. Fully naturalistic settings may yield additional insights. Furthermore, medication use was not controlled for and may have influenced attentional and emotional performance, yet the observed patterns remained robust across participants. Cultural and linguistic factors may also influence emotion recognition and attentional patterns, suggesting the need for cross-cultural validation.

Future research should explore longitudinal designs to track developmental trajectories and evaluate the long-term effects of interventions on attentional and emotional regulation, particularly regarding their applications and potential for personalized intervention plans, thereby reinforcing the clinical relevance of combining behavioral, emotional, and neurocognitive indices.

### Conclusion

The findings indicate that children with lower attentional performance exhibit a complex interplay of attentional instability, oculomotor variability, and heightened emotional reactivity, particularly in response to anger and fear stimuli. These patterns support the proposed hypotheses and suggest that attentional functioning is dynamically shaped by interactions between cognitive and emotional processes. The results highlight the multidimensional nature of attentional functioning, extending beyond isolated cognitive mechanisms to include integrated emotional dynamics. Notably, variability across oculomotor and emotional domains suggests that attentional performance is better understood as emerging from interactions between these systems rather than as discrete abilities.

From a clinical and educational perspective, the multimodal approach, grounded in real-time data capture and post hoc interpretable ML analysis, yields objective, scalable indicators of attentional and emotional functioning, providing a basis for generating hypotheses about future personalized intervention strategies.

This study provides preliminary evidence supporting the use of a multimodal screening approach for differentiating children with varying levels of attentional performance. By integrating eye-tracking, facial emotion recognition, and neuropsychological measures within a single platform, the random forest model achieved high predictive accuracy and robust generalization, as confirmed by nested cross-validation, highlighting its potential as a complementary and ecologically valid assessment tool. The ability to capture oculomotor and emotional data simultaneously within a single session supports the feasibility of a practical, time-efficient assessment approach, though further validation in pediatric and educational settings is needed. Additionally, the use of interpretable ML techniques (eg, SHAP) enhances model transparency and applicability across research and applied contexts. Overall, integrating multimodal behavioral data within an explainable AI framework represents a promising direction for advancing assessment practices regarding attentional functioning across development.

## Data Availability

The data that support the findings of this study are available from the corresponding author, MGP, upon reasonable request.

## Appendix

Multimedia Appendix 1 All 30 selected features ranked by mean absolute Shapley additive explanations value.

## Abbreviations

ADHD: attention-deficit/hyperactivity disorder
AOI: area of interest
AUC: area under the curve
AUC-CV: cross-validated area under the curve
AUC-ROC: area under the receiver operating characteristic curve
CNAB: Coimbra Neuropsychological Assessment Battery
EF: executive function
FN: false negative
FP: false positive
LightGBM: light gradient boosting machine
ML: machine learning
SHAP: Shapley additive explanations
SVM: support vector machine
TMT: Trail Making Test
XGBoost: extreme gradient boosting
