# Supplementary material for: Neurobiosensory and Visual Patterns of Attentional and Emotional Functioning in Children Using Machine Learning: Cross-Sectional Study
Source: JMIR Pediatr Parent. 2026 Aug 10;9:e90041. doi: 10.2196/90041 (PMC13504248; doi:10.2196/90041)
Supplement: Supplementary file 1 [file pediatrics_v9i1e90041_app1.docx]

*Supplementary Table: All 30 selected features ranked by mean absolute SHAP value. |SHAP| = mean absolute SHAP (higher = more influential). MI = Mutual Information score. Direction = which class the feature pushes toward.*

| **#** | **Feature** | **\|SHAP\|** | **MI** | **Group** | **Explanation** | **Clinical Interpretation** |
| --- | --- | --- | --- | --- | --- | --- |
| 1 | recognition_bias_mean | 1.7263 | 0.0842 | Aggregated Emotion | Ratio between fixations on negative vs. positive emotional faces. | High values indicate attentional bias toward negative/threatening stimuli. |
| 2 | gaze_to_joy_mean | 1.2491 | 0.0602 | Per-Emotion / Gaze | Proportion of fixations on joyful faces. | Altered allocation to positive faces may reflect atypical social processing. |
| 3 | visual_efficiency_mean | 1.0888 | 0.0251 | Eye-tracking | Proportion of fixations directed to informative regions (eyes, mouth, face). | High values suggest excessive fixation on limited regions rather than adaptive exploration. |
| 4 | expr_dur_fear_mean | 0.7529 | 0.0513 | Per-Emotion / Expression | Mean duration of fear-expression episodes. | Longer fear episodes suggest sustained anxiety-related responses. |
| 5 | expr_time_sadness_mean | 0.7167 | 0.0318 | Per-Emotion / Expression | Proportion of time expressing sadness. | Higher values indicate prolonged negative affective state. |
| 6 | low_confidence_mean | 0.6364 | 0.0260 | Aggregated Emotion | Frames where facial-expression detection confidence was low. | May reflect unstable or diffuse emotional expression. |
| 7 | expr_dur_anger_mean | 0.5714 | 0.0521 | Per-Emotion / Expression | Mean duration of anger-expression episodes. | Longer anger episodes suggest difficulty in emotional regulation. |
| 8 | gaze_path_length_mean | 0.2755 | 0.3499 | Eye-tracking | Total normalized gaze-path length across the task. | Longer gaze paths indicate less focused exploration. |
| 9 | speed_cv_mean | 0.2743 | 0.3198 | Eye-tracking | Variability of oculomotor speed. | High variability reflects unstable oculomotor control. |
| 10 | gaze_dispersion_mean | 0.2460 | 0.3284 | Eye-tracking | Spatial dispersion of gaze coordinates. | Higher dispersion suggests reduced attentional focus. |
| 11 | cv_fix_duration_mean | 0.2178 | 0.0960 | Eye-tracking | Variability of fixation durations. | High variability indicates inconsistent sustained attention. |
| 12 | ifi_cv_mean | 0.1962 | 0.0808 | Eye-tracking | Variability of inter-fixation intervals. | Irregular fixation rhythm associated with attentional instability. |
| 13 | IIA_std | 0.1802 | 0.0443 | Composite Index | Variability of Attentional Instability Index across tasks. | High variability reflects inconsistent attentional control. |
| 14 | mean_fix_duration_mean | 0.1770 | 0.0934 | Eye-tracking | Mean fixation duration. | Longer fixations may indicate inefficient processing. |
| 15 | emotion_transition_rate_mean | 0.1654 | 0.0777 | Aggregated Emotion | Rate of transitions between facial emotions. | Frequent transitions indicate emotional instability. |
| 16 | Age | 0.1641 | 0.1608 | Demographic | Chronological age in years. | Younger age associated with higher attentional error baseline. |
| 17 | emotional_intensity_mean | 0.1624 | 0.0812 | Aggregated Emotion | Mean intensity of dominant facial emotion. | High intensity suggests elevated emotional reactivity. |
| 18 | zone_switch_rate_mean | 0.1562 | 0.3121 | Eye-tracking | Frequency of gaze switching between visual zones. | High switching reflects agitated visual scanning. |
| 19 | n_active_emotions_mean | 0.1539 | 0.0936 | Aggregated Emotion | Number of distinct emotions expressed. | Greater emotional fragmentation and instability. |
| 20 | gaze_to_anger_mean | 0.1318 | 0.0454 | Per-Emotion / Gaze | Proportion of fixations on angry faces. | Indicates attentional bias toward threat-related stimuli. |
| 21 | dur_emotion_bias_mean | 0.1226 | 0.0460 | Aggregated Emotion | Difference between time spent in negative vs. positive emotions. | Greater negative emotional predominance associated with attentional vulnerability. |
| 22 | expr_std_fear_mean | 0.1081 | 0.0549 | Per-Emotion / Expression | Variability of fear expression. | Reflects unstable fear-related emotional responses. |
| 23 | expr_joy_mean | 0.0893 | 0.0425 | Per-Emotion / Expression | Mean intensity of joy expression. | Lower joy expression suggests reduced positive engagement. |
| 24 | ICO_mean | 0.0826 | 0.0356 | Composite Index | Gaze–Emotion Congruence Index. | Lower congruence reflects weaker emotional-attentional integration. |
| 25 | IRE_mean | 0.0800 | 0.0271 | Composite Index | Emotional Reactivity Index. | Higher values indicate stronger emotional dysregulation/reactivity. |
| 26 | valence_balance_mean | 0.0748 | 0.0661 | Aggregated Emotion | Balance between negative and positive emotions. | Greater negative valence associated with frustration and dysregulation. |
| 27 | IIA_mean | 0.0466 | 0.0778 | Composite Index | Attentional Instability Index. | Higher values indicate fragmented attentional profile. |
| 28 | expr_time_surprise_mean | 0.0268 | 0.0742 | Per-Emotion / Expression | Time expressing surprise. | Prolonged surprise may indicate slower processing/disorientation. |
| 29 | expr_gaze_congruence_mean | 0.0218 | 0.0348 | Per-Emotion / Expression | Congruence between expressed emotion and gaze target. | Lower congruence suggests atypical emotional processing. |
| 30 | fixation_quality_mean | 0.0000 | 0.0384 | Eye-tracking | Composite fixation-quality index. | Lower fixation quality associated with unstable visual attention. |
